# Thrombocytopenia in the intensive care unit

**DOI:** 10.12669/pjms.35.1.19

**Published:** 2019

**Authors:** Zohreh Ostadi, Kamran Shadvar, Sarvin Sanaie, Ata Mahmoodpoor, Seied Hadi Saghaleini

**Affiliations:** 1 Zohreh Ostadi, Anesthesiologist, Fellowship of Critical Care Medicine, Department of Anesthesiology, Faculty of Medicine, Tabriz University of Medical Sciences, Tabriz, Iran; 2 Kamran Shadvar, Associate Professor of Anesthesiology, Fellowship of Critical Care Medicine, Department of Anesthesiology, Faculty of Medicine, Tabriz University of Medical Sciences, Tabriz, Iran; 3 Sarvin Sanaie, Assistant Professor of Nutrition, MD, PhD, Tuberculosis and Lung Disease Research Center, Tabriz University of Medical Sciences, Tabriz, Iran; 4 Ata Mahmoodpoor, Professor of Anesthesiology, Fellowship of Critical Care Medicine, Department of Anesthesiology, Faculty of Medicine, Tabriz University of Medical Sciences, Tabriz, Iran; 5 Seied Hadi Saghaleini, Assistant Professor of Anesthesiology, Fellowship of Critical Care Medicine, Department of Anesthesiology, Faculty of Medicine, Tabriz University of Medical Sciences, Tabriz, Iran

**Keywords:** Thrombocytopenia, Intensive care unit, Pathogenesis, Management

## Abstract

Thrombocytopenia is a frequent finding in intensive care unit especially among adults and medical ICU patients. Thrombocytopenia is defined as a platelet count less than 100×10^9^/l in ICU setting. Platelets are made in the bone marrow from megakaryocytes. Although not fully understood, proplatelets transform into platelets in the lung. The body tries to maintain platelet count relatively constant throughout life. Pathophysiology of thrombocytopenia can be defined by hemodilution, elevated levels of platelet consumption, compromise of platelet production, increased platelet sequestration and increased platelet destruction. Unlike in other situations, absolute platelet count alone does not provide sufficient data in characterizing thrombocytopenia in ICU patients. In such cases, the time course of changes in platelet count is also pivotal. The dynamics of platelet count decrease vary considerably between different ICU patient populations including trauma, major surgery and minor surgery/medical conditions.

There are strong evidences available that delay in platelet count restoration in ICU patients is an indicator of a bad outcome.

## INTRODUCTION

Thrombocytopenia is a frequent finding in intensive care unit (ICU), with an incidence ranging from 13% to almost 60% in different studies. This variability is due to the vulnerability of different patients to thrombocytopenia. For example, it has been shown that thrombocytopenia is more frequent among adults and medical ICU patients in comparison with children and surgical ICU cases. In addition, thrombocytopenia is not apparent at the time of ICU admission in many cases, while it develops in many patients while they stay in the ICU.^1^,^2^

There is inconsistency regarding the clinical significance of thrombocytopenia in ICU setting. While many authors believe that the emergence of thrombocytopenia in ICU patients could dramatically increase the risk of morbidity and mortality (usually because of an increased risk of bleeding); others suggest that this thrombocytopenia is only an indicator of another more important underlying cause, such as a critical illness or using a particular medication. The present article tries to briefly summarize all essential information regarding thrombocytopenia in ICU setting and accordingly increase the level of knowledge and awareness of physicians who deals with ICU patients

### Normal production and activation of platelets

Platelets are made in the human bone marrow from megakaryocytes. Although not fully understood, it has been proposed that proplatelets transform into platelets in the lung after release from the bone-marrow.^3^ The body tries to maintain platelet count relatively constant throughout life.^4^ This regulation is done by secreting thrombopoietin (TPO), a hormone produced by hepatic and renal cells and affects the megakaryocytes to multiply and differentiate to platelets.^5^

The lifespan of a normal platelet is 8-10 days in the blood stream, and almost on third of the total platelet mass is located in the spleen.^6^ When the endovascular lining is injured, the collagen fibrils are exposed to circulating platelets and clot formation is induced after activation of a coagulation cascade. When a platelet is activated, it releases thromboxane A2 and adenosine diphosphate, which in turn attract and activate further platelets. Activated platelets also bind to the glycoprotein IIb/IIIa receptor placed on circulating fibrinogen which induce local aggregations of platelets and fibrin deposition.^7^

### Definition and incidence of thrombocytopenia

Thrombocytopenia is defined as a platelet count less than 150×10^9^/l. In ICU setting, the defined cut-off point has been lowered to 100×10^9^/l by some authors, with <50×10^9^/l suggested as severe thrombocytopenia with surgical bleeding.^8^ A spontaneous bleeding usually occurs in patients with a platelet count <10000/ml.^9^

Unlike in other situations, absolute platelet count alone does not provide sufficient data in characterizing thrombocytopenia in ICU patients. In such cases, the time course of changes in platelet count is also pivotal. For example, a decline more than 50% compared to the baseline value is considered normal after cardiac surgery, but it is abnormal in the second week of ICU stay. It is also true in patients with no rise in platelet count within five days after ICU admission. So, it is essential to use both absolute platelet count and its changes during ICU stay to define thrombocytopenia.^10^

As stated before, the incidence of thrombocytopenia in ICU patients ranges between 13% and 60% in various studies^1^,^2^; around 20% in medical ICU patients,^11^ 35% in surgical ICU patients,^12^ and 45% in trauma ICU patients.^13^

### Pathophysiology and etiologies of thrombocytopenia

Pathophysiology of thrombocytopenia, in general, can be defined by five mechanisms: hemodilution^14^, elevated levels of platelet consumption^15^, compromise of platelet production^16^, increased platelet sequestration^17^, and increased platelet destruction. The first two mechanisms are considered as the major etiologies of thrombocytopenia in ICU patients^10^,^18^ (Table-I).

**Table-I:** Mechanisms of thrombocytopenia.

Mechanism	Example	Description
Hemodilution		Massive transfusions of blood products due to massive hemorrhage
Increased platelet consumption	DIC, TTP, HELP syndrome	
Decreased platelet production	Chemotherapy, radiotherapy, cancer, transient viral infections (mumps, rubella, varicella), chronic infections (chronic hepatitis C infection, HIV infection), alcohol abuse, sepsis, drugs, malnutrition, storage disorders, decreased thrombopoietin	Bone marrow failure or disease, miscellaneous
Increased platelet sequestration	Cirrhosis, portal hypertension, polycythemia vera, infections, congestive heart failure	Because of splenomegaly
Increased platelet destruction	HIT, ITP, mechanical devices, microangiopathies, drugs, splenomegaly, sepsis	Immune-mediated, miscellaneous

DIC: disseminated intravenous coagulopathy, HELP: hemolysis, elevated liver enzymes, low platelets, HIT: heparin-induced thrombocytopenia, HIV: human immunodeficiency virus, ITP: idiopathic thrombocytopenic purpura, TTP: thrombotic thrombocytopenic purpura

Another important issue in evaluation of thrombocytopenia in ICU patients is the possibility of pseudothrombocytopenia. Using medications with glycoprotein IIb/IIIa antagonistic properties such as abciximab, integrilin or tirofiban may also contribute to this condition. In such cases, platelet aggregates are present in peripheral blood smears.^19^ Repeated blood counts in citrated or heparinized blood could rule out idiopathic pseudothrombocytopenia.^20^

Various etiologies may underlie the occurrence of thrombocytopenia in ICU patients, including drug exposure (Table-II), intravascular devices (such as pulmonary artery catheters, intra-aortic balloon pumps, central venous catheter), higher severity of illness score in ICU admission^21^, sepsis, and hypothermia.^22^

**Table-II:** Important drugs contributing to thrombocytopenia in ICU patient population.

Name	Possible mechanism(s)
Aspirin	Inhibits cyclooxygenase 1, blocks thromboxane A2 production, disrupts platelet aggregation
Nonsteroidal anti-inflammatory drugs	Inhibit cyclooxygenase 1
Clopidogrel	Blocks glycoprotein IIb/IIIareceptor, induces thrombotic thrombocytopenic purpura
Dipyridamole and ticlopidine	Block glycoprotein IIb/IIIa receptor
Abciximab	Glycoprotein IIb/IIIa receptor antagonist
Eptifibatide	Glycoprotein IIb/IIIa receptor antagonist, displaces fibrinogen from activated glycoprotein IIb/IIIa receptors, facilitates dispersal of platelet aggregates
Antibiotics (trimethoprim/sulfamethoxazole, blactam antibiotics, vancomycin, linezolid)	Unknown, miscellaneous
Anticonvulsive drugs (phenytoin, carbamazepine, valproate, phenobarbital)	Unknown, miscellaneous
Antihistamines (ranitidine)	Unknown, miscellaneous

### Dynamics of normal platelet count decrease in ICU patients

The dynamics of platelet count decrease vary considerably between different ICU patient populations including trauma, major surgery and minor surgery/medical conditions.^10^

In major postsurgical cases a nadir is typically seen between day one and day four. This decrease is usually due to platelet consumption and correlates significantly with the extent of tissue injury and blood loss. In normal conditions, the platelet count reaches back to a normal level two weeks after surgery.^23^ In abdominal surgery patients, however, the nadir occurs earlier (day 1-2) and the recovery is faster (day 3-4).^24^

In traumatic cases with critical injuries, the admission platelet count is usually normal and a rapid platelet count decrease happens within hours after hospitalization.^25^ A low platelet count at admission, and delay in recovery during ICU stay has been regarded as an ominous sign.^26^

In medical patients, the dynamics of platelet count changes depend greatly on the underlying condition. For example, sepsis, endocarditis, leukemia, thromboembolism, chronic infections, alcohol intoxication, using intravascular devices, renal replacement therapy, employment of extracorporeal circuits, disseminated intravenous coagulopathy (DIC), multiorgan failure and late cardiopulmonary resuscitations are in essence associated with thrombocytopenia.^27^-^30^

Generally, any rapid decrease in platelet count within 24-48 hours after ICU admission in any population, or when a platelet count increment is less than 5000/ml one-hour post transfusion (refractoriness) an immune-mediated process that destroy transfused platelets rapidly (such as ITP, alloimmunization, heparin or any other drug-induced immune thrombocytopenia) is suggested, whereas a gradual decline indicates marrow suppression of thrombopoiesis or subacute platelet consumption.^27^

The nadir in drug-induced thrombocytopenia is typically less than 10000/µl, while in heparin-induced cases the nadir is usually more than 20000-50000/µl. Table-III summarizes differential diagnosis of thrombocytopenia in ICU patients.

**Table-III:** Differential diagnosis of thrombocytopenia in the ICU.

Differential diagnosis	Findings
Sepsis	Presence of dedicated criteria such as positive cultures and clinical findings
Disseminated intravascular coagulation	Abnormal laboratory findings
Massive blood loss	Abnormal laboratory findings, bleeding
Thrombotic microangiopathy	Schistocytes in blood examination, clinical findings
Heparin-induced thrombocytopenia	A positive history of heparin use, laboratory findings
Immune thrombocytopenia	Immunological and laboratory findings
Drug-induced thrombocytopenia	Abnormal findings in bone aspiration, immunological and laboratory findings

### DIT/HIT

Drug-induced thrombocytopenia in ICU patients can be divided as non-immune (such as histamine H_2_ antagonists, nonsteroidal anti-inflammatory drugs,) and immune (such as IIb/IIIa inhibitors, trimethoprim/sulfamethoxazole, vancomycin, penicillin, ceftriaxone, mirtazapine, ibuprofen and unfractionated heparin) categories.^30^ Heparin-induced thrombocytopenia (HIT) is caused by the development of IgG antibodies directed against a complex of platelet factor 4 (PF4) and heparin^31^ and presenting with more than a half of the baseline platelet count, to 50-80×10^9^/L or a new thrombosis occurring 5-14 days after heparin initiation.^30^ Table-IV shows 4T score for prediction of HIT in critically ill patients.

**Table-IV:** 4T score for prediction of HIT in critically ill patients.

Points*	2	1	0
Thrombocytopenia	>50% fall andplatelet minimum	30–50% fall or platelet minimum 10–19 × 10^9^/l	Fall <30% or platelet minimum<10 × 10^9^/l
	≥ 20 × 10^9^/l		
Timing** of platelet count fall or other sequelae	Clear onset between days 5 and 10; or ≤1d (if heparinexposure within past30 d)	Consistent with immunization but not clear or onset of thrombocytopenia after day 10; or fall ≤1d (if heparin exposure30–100 d ago)	Platelet count fall ≤4 d(without recent heparin exposure)
Thrombosis or other sequelae	New thrombosis; skin necrosis; post- heparin bolus acute systemic reaction	Progressive or recurrent thrombosis; erythematous skin lesions; suspected thrombosis not yet proven	None
Other cause for thrombocytopenia not evident	No other cause is evident	Possible other cause is evident	Definite other cause is present

* (0, 1, or 2 for each category; maximum score = 8) Pretest probability score: 6–8 = High; 4–5 = Intermediate; 0–3 = Low.

** First day of immunizing heparin exposure considered day 0; the day the platelet count begins to fall is considered the day of onset of thrombocytopenia

### Management of thrombocytopenia in different ICU settings

In trauma cases, coagulopathy is due to a mixture of underlying pathologies such as accelerated platelet/clotting factors consumption, hemodilution, metabolic abnormalities, hypothermia, etc. that need to be corrected concomitantly. Early administration of plasma, platelet and red blood cells has been found vital in a recent study.^32^ Using antifibrinolytic agents such as tranexamic acid is also suggested in severe trauma patients.^33^ When DIC is present, platelet transfusion should be reserved for cases with active bleeding and in those who are diagnosed as very high risk for bleeding.^34^ In septic patients there is a controversy in transfusing platelet.^35^ Bone marrow suppression is usually along with pancytopenia rather than an isolated thrombocytopenia that rarely develops independently in ICU patients because of an unrecognized comorbidity such as malignancy, infection, osteoporosis, myelofibrosis, etc.^10^,^36^-^37^ Specific treatments such as charcoal ingestion, dialysis and specific antidote use are necessary.^38^ Early plasma exchange with or without corticosteroid/rituximab administration is indicated in such patients.^39^ Hemolytic-uremic syndrome (HUS) should be managed by using monoclonal humanized anti-C5 antibody, eculizumab andimmunoadsorption.^37^ Antiphospholipid syndrome may be present in ICU patients and should be managed by anticoagulation, platelet inhabitation, plasma exchange, immunoadsorption, corticosteroids, and intravenous immunoglobulins.^38^ Long-term immunosuppression with cyclophosphamide or rituximab and eculizumab may also be necessary.^38^ If sever bleeding is present, intravenous immunoglobulin (1 g/kg for 2 days) and platelet transfusion are indicated. In case of a definite diagnosis, heparin use should be ceased and replaced by an alternative non-heparin anticoagulant, platelet count monitoring should be performed every two or three days from day four to day 14, platelets should not be given for prophylaxis but may be used in the event of bleeding, patients should be therapeutically anticoagulated for 3 months after HIT with a thrombotic complication and for 4 weeks following HIT without a thrombotic complication.^39^ Table-V shows treatment recommendations for patients with HIT.

**Table-V:** Treatment recommendations for patients with HIT.

• Clinical decisions should be made following consideration of the risks and benefits of treatment with an alternative anticoagulant
• For patients with suspected or confirmed HIT, heparin should be stopped and full dose anticoagulation with an alternative anticoagulant commenced
• LMWH should not be used in the treatment of HIT
• Warfarin should not be used until the platelet count has recovered to the normal range. When introduced, an alternative anticoagulant must be continued until the INR is therapeutic. Argatroban affects the INR and this needs to be considered when using this drug. A minimum overlap of 5 d between non-heparin anticoagulants and VKA therapy is recommended
• Platelets should not be given for prophylaxis but may be used in the event of bleeding
• If the patient has received a VKA at the time of diagnosis it should be reversed by administering intravenous vitamin K

### Platelet transfusion

There is no fixed platelet count threshold in ICU patients that signals platelet transfusion,^32^ but because of hemorrhage fear there is a widespread notion that the platelet count should be maintained over 100×10^9^/L in massive bleeding or when bleeding occurs at dangerous sites such as in intracranial hemorrhage.^33^

In addition to platelet count, the risk of hemorrhage is also dependent on the hematocrit and the bleeding time.^34^ So, red blood cell transfusion should also be considered as a part of a supportive therapy, if needed.^10^

Despite these recommendations, it should be born in mind that platelet transfusion is not a risk-free procedure and may cause several potentially fatal complications such as contamination, allergic reactions, venous thromboembolism and transfusion-related acute lung injury (TRALI).^35^ Another problem in ICU patients is bleeding in connection with antiplatelet drug use. In such cases, platelet transfusion is usually considered as an antidote, but circulating metabolites of antiplatelet drugs inhibit transfused platelets, either.^36^

An efficient platelet transfusion in ICU setting could be judged by cessation/reduction of clinical bleeding. Unlike in non-ICU patients, this judgment might be very difficult in ICU cases because of the complexity of patients’ condition owing to the presence of a myriad of concomitant cofactors that could be hardly controlled.^10^ Factors that negatively affect the increase in platelet count after platelet transfusions are using stored platelets for a long-term, fever/infection, and presence of splenomegaly in the recipient.^40^

### Prognosis of thrombocytopenia in ICU

It has been shown that the development of thrombocytopenia during an ICU admission significantly increases morbidity and mortality.^41^ According to these information, morbidity and mortality rates have been more striking in patients who develops thrombocytopenia after ICU admission compared to patients whose platelet count is normalized or remains stable within the first week of ICU stay. The prognosis has been found even worse in patients whose thrombocytopenia worsened or not resolved beyond the first four to seven days of ICU admission.

### Author’s Contribution

**ZO:** Literature review, writing the initial draft of the manuscript and approving final version.

**KSh:** Literature review and approving final version.

**SS:** Drawing tables, editing language and approving final version.

**AM:** Concept of manuscript, Literature review, editing and approving final version.

**SHS:** Literature review and approving final version.
